# Supplementary material for: Optimising the balance of acute and intermediate care capacity for the complex discharge pathway: Computer modelling study during COVID-19 recovery in England
Source: PLoS One. 2022 Jun 7;17(6):e0268837. doi: 10.1371/journal.pone.0268837 (PMC9173611; doi:10.1371/journal.pone.0268837)
Supplement: S1 File — (DOCX) [file pone.0268837.s001.docx]

**Supporting Information 1: Pathway simulation model**

The pathway computer simulation model developed for the purposes of this study aims at determining required capacity for the three D2A step-down intermediate care pathways independently (P1, P2 and P3). The model was coded in R (version 3.6.4) as an initial improvement to a Microsoft Excel based deterministic model used for capacity planning by the collaborating health and care organisations. All simulation parameters were estimated in collaboration with the healthcare services involved. Figure 1 (main paper) illustrates the general flow of the patients simulated in this model from arrival and stay in hospital, to discharge into P1-3 pathways.

From a modelling perspective, there are two types of pathways depending on the type of service patients require and the related capacity involved: visit-based and bed-based. Patients entering a visit-based care pathway (P1) reside in their usual place of residence and community care is provided by one or more care workers via daily visits. For this type of pathway, capacity is based on the number of (time) slots and the required number of visits per slot. Patients needing bed-based care (P2 and P3) will transfer to a step-down facility after discharge from the acute hospital for a pre-determined length of stay, hence the capacity is evaluated in terms of the number of community health beds required.

Costs used in the model are presented in Table SI.1.2. As the focus of this paper is the identification of cost-optimal intermediate care capacity through minimising system costs, we chose to use relative, rather than absolute, costs. Plots 3 (main paper), SI.2.1, SI.2.2 and SI.2.3 display all costs indexed to the minimum (‘cost-optimum’) of the baseline scenario (Scenario 1) for each pathway.

***The visit-based care pathway model***

Daily referral rates into P1 were generated using a Poisson process where the mean for each day has been estimated using the prediction models described in subsection ‘Demand projection’ of the main paper. Patients who are referred but unable to be discharged into P1 remain in hospital and represent a delayed discharge. Reflecting current data, the duration of P1 service is sampled from a normal distribution. The initial daily visit requirements, and the final daily visit requirements are sampled from normal distributions. The mean and standard deviation of the baseline duration of service were estimated from up-to-date data provided by intermediate care providers. Although, the number of visits required per patient-day is not recorded on a regular basis, the intermediate care service base their planning on an average of three care visits per day. In practice, it is known that the number of visits tapers over the duration of service. Also, patients in P1 may initially require two care staff per visit. Accordingly, we set the mean of initial number of visits required to four (truncated to an upper limit of six) and the mean of the final number of visits to two. In the model, a sequence of visits from the sampled initial number to the sampled final number of visits across a duration of service was generated per simulated patient. For example:

- If the initial number of visits is sampled as six, and
- the final number of visits is sampled as two, and
- the duration of service is sampled as 10 days,
- then, the visit sequence for the patient is [6,6,5,5,4,4,3,3,2,2].

The capacity is the number of visits in the system, i.e., the number of patients admitted into P1 (‘slots’), multiplied by the average number of visits per day. Patients with a long duration of service, and/or a high initial/end visit requirement subsequently may prevent patients with lower service requirements from entering the P1 system. For this reason, if there are no available resources to start service for a new patient immediately, an arriving patient is scheduled to start on the following day. A patient whose visit sequence can be integrated into the available P1 capacity will be scheduled immediately.

***The bed-based care pathway model***

As in the visit-based model, daily referrals were generated using a Poisson distribution with daily means estimated from the demand prediction models described in in subsection ‘Demand projection’ of the main paper. For each patient, the length of stay was sampled from a lognormal distribution with parameters estimated using the data obtained from the CCG. Any patient who is referred but unable to be transferred into the assigned bedded pathway will remain in hospital as a delayed discharge. In this model, the capacity is represented by the number of beds available and any patient who enters the corresponding pathway occupies the bed until the end of their length of stay. The maximum capacity needed is represented by the number of beds required to have zero delayed discharges from the acute hospital. To reflect the current situation in the considered system each bed-based care pathway, in this case P2 and P3, is treated independently.

The input parameter values used in the pathway simulation model are presented in Table SI.1.1.

**Table SI.1.1.** Pathway simulation model input parameters (LoS: Length of Stay). ^*^ Estimated from care provider data.

| Pathway | Number of simulation runs (replications) | Initial occupancy on 14 May 2021^*^ | Mean LoS^*^ | LoS Distribution | Mean Arrival Rate | Arrivals  Distribution | Proportion of patients entering pathway (%) ^*^ |
| --- | --- | --- | --- | --- | --- | --- | --- |
| P1 | 200 | 184 | 13 | Normal | Daily projection | Poisson | 54 |
| P2 | 200 | 151 | 29 | Lognormal | Daily projection | Poisson | 20 |
| P3 | 200 | 172 | 43 | Lognormal | Daily projection | Poisson | 17 |

The cost data used to simulate system costs are presented in Table SI.1.2.

**Table SI.1.2.** Simulation model cost parameters, cost ratios, and sources.

|  | Average cost of weekly service | Relative Cost ratios | Source of costs |
| --- | --- | --- | --- |
| P1 | £875 | 5 | 2017/18 NHS reference costs |
| P2 | £1,050 | 6 | National Audit of Intermediate Care 2017/18 |
| P3 | £1,150 | 7 | System costs from Bristol care system |
| Acute | £2422 | 14 | 2017/18 NHS reference costs |

To determine the optimum capacity which minimizes the total cost of acute delayed discharges and the cost of providing surplus capacity, the simulation model calculated the overall cost across a range of feasible capacities. The capacity is either the number of visits or the number of beds available to the system. To determine the lower and upper bound of the range of capacity required, the simulation was first run assuming infinite available capacity to find the capacity needed for zero acute hospital delayed discharges. The upper bound was set to 75% quantile of the maximum capacity needed. A lower bound that reflects the evolution of the costs with respect to capacity was set by visual inspection of the outputs. The total daily cost we considered includes the delayed discharge cost in the acute and the service cost in intermediate care. The average cost of delayed discharge in acute care is calculated as the cost of one hospital day multiplied by the average delay per simulated patient and the number of patients discharged per week.
