# Supplementary material for: Optimising the balance of acute and intermediate care capacity for the complex discharge pathway: Computer modelling study during COVID-19 recovery in England
Source: PLoS One. 2022 Jun 7;17(6):e0268837. doi: 10.1371/journal.pone.0268837 (PMC9173611; doi:10.1371/journal.pone.0268837)
Supplement: S2 File — (DOCX) [file pone.0268837.s002.docx]

**Supporting Information 2: Sensitivity analysis accounting for uncertainty regarding age and multimorbidity of acute admissions**

In our baseline model we assumed that 19% of acute discharges are complex and patients enter one of the three community care pathways after discharge. However, we recognise that this figure may vary based on different patient-related characteristics such as age, presence of chronic conditions and/or comorbidities. In order to account for this uncertainty, we present a sensitivity analysis over the proportion of complex discharges by running simulation assuming a complex discharge proportion of 10% (Figures SI.2.1 and SI.2.2) and 30% (Figures SI.2.3 and SI.2.4).


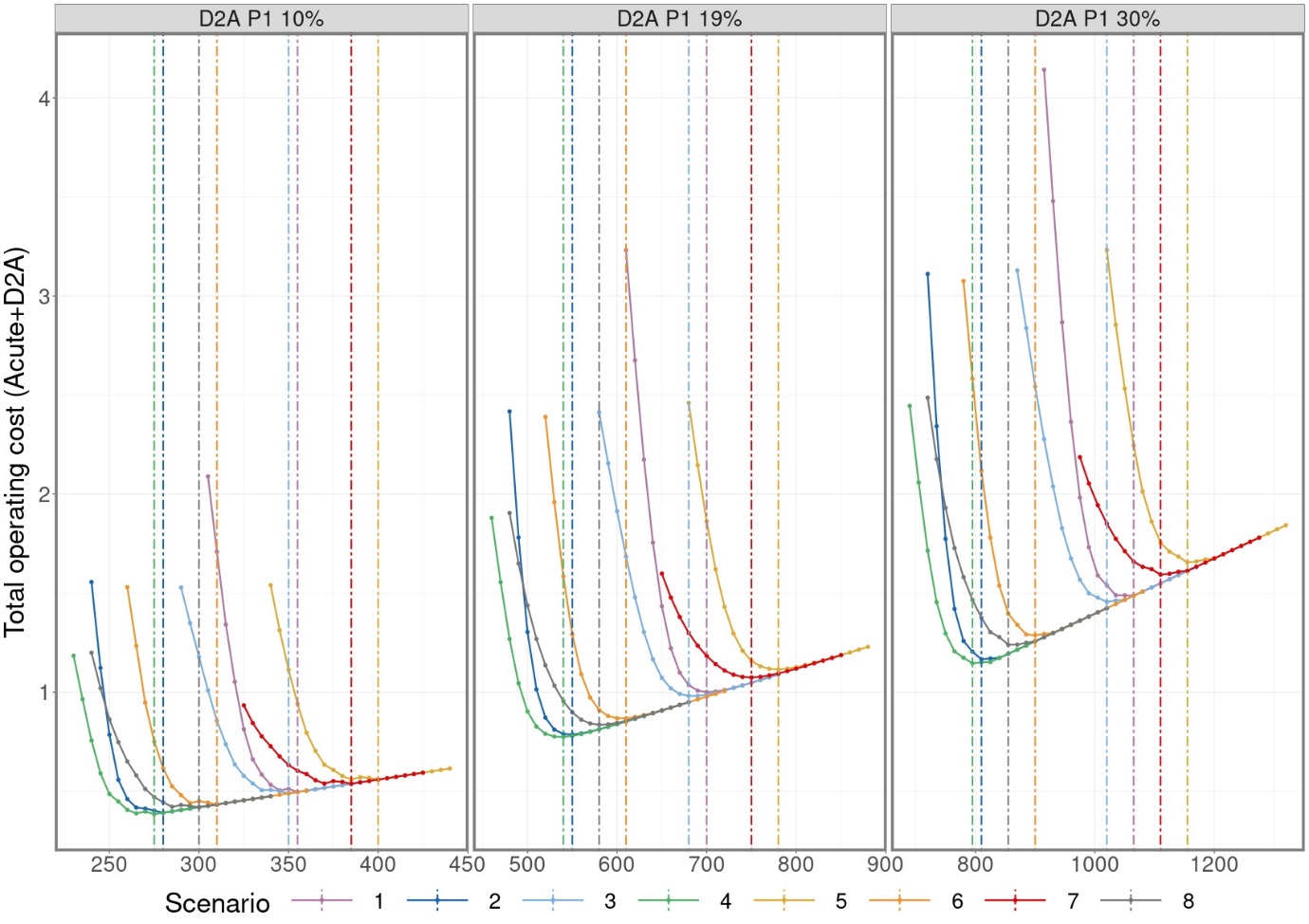


**Figure SI.2.1.** Weekly cost of acute delay and pathway care community service for capacity configurations for Pathway 1 with 10%, 19% (baseline) and 30% of acute admissions requiring complex community services at discharge.

**
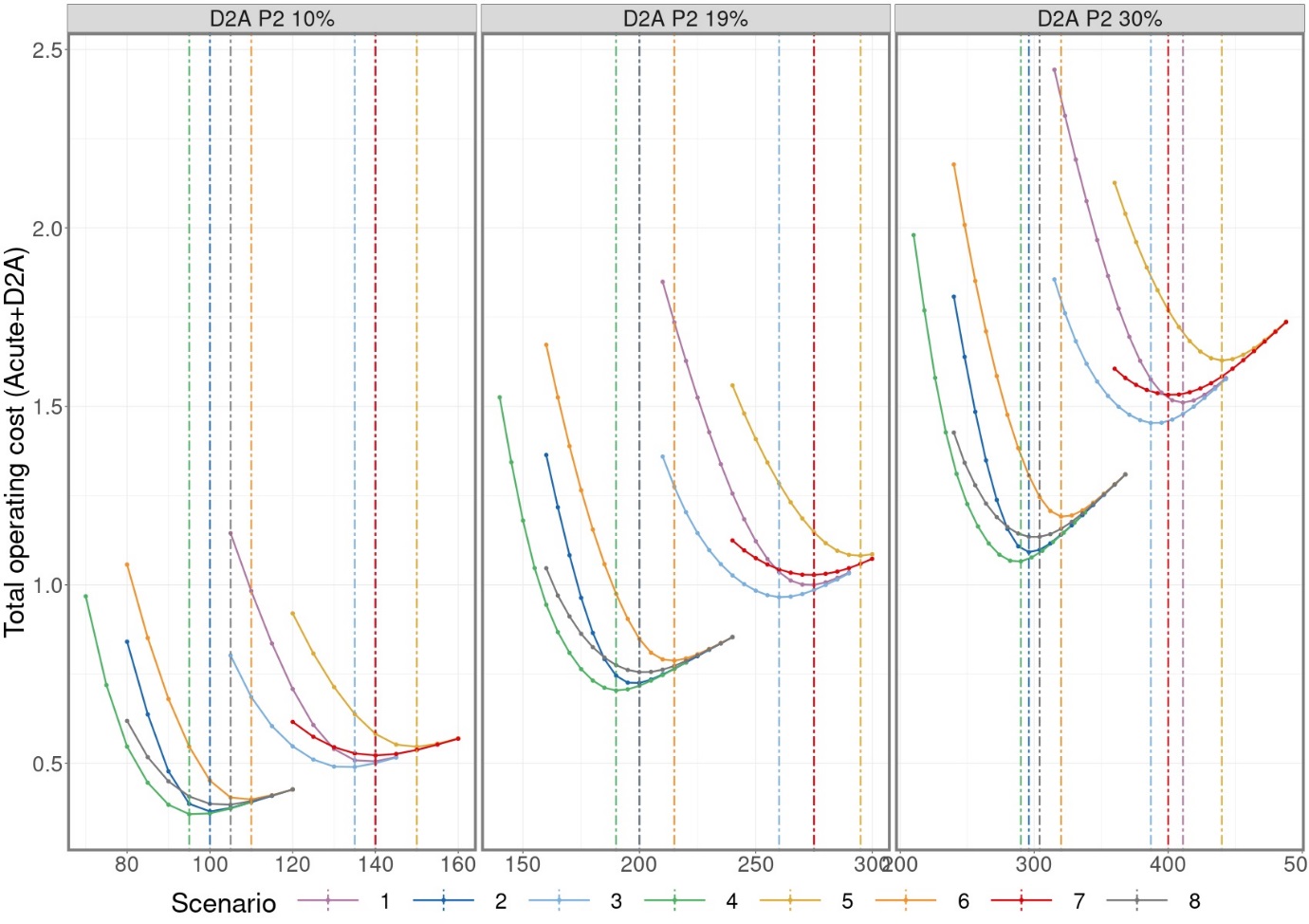
**

**Figure SI.2.2.** Weekly cost of acute delay and pathway care community service for capacity configurations for Pathway 2 with 10%, 19% (baseline) and 30% of acute admissions requiring complex community services at discharge.


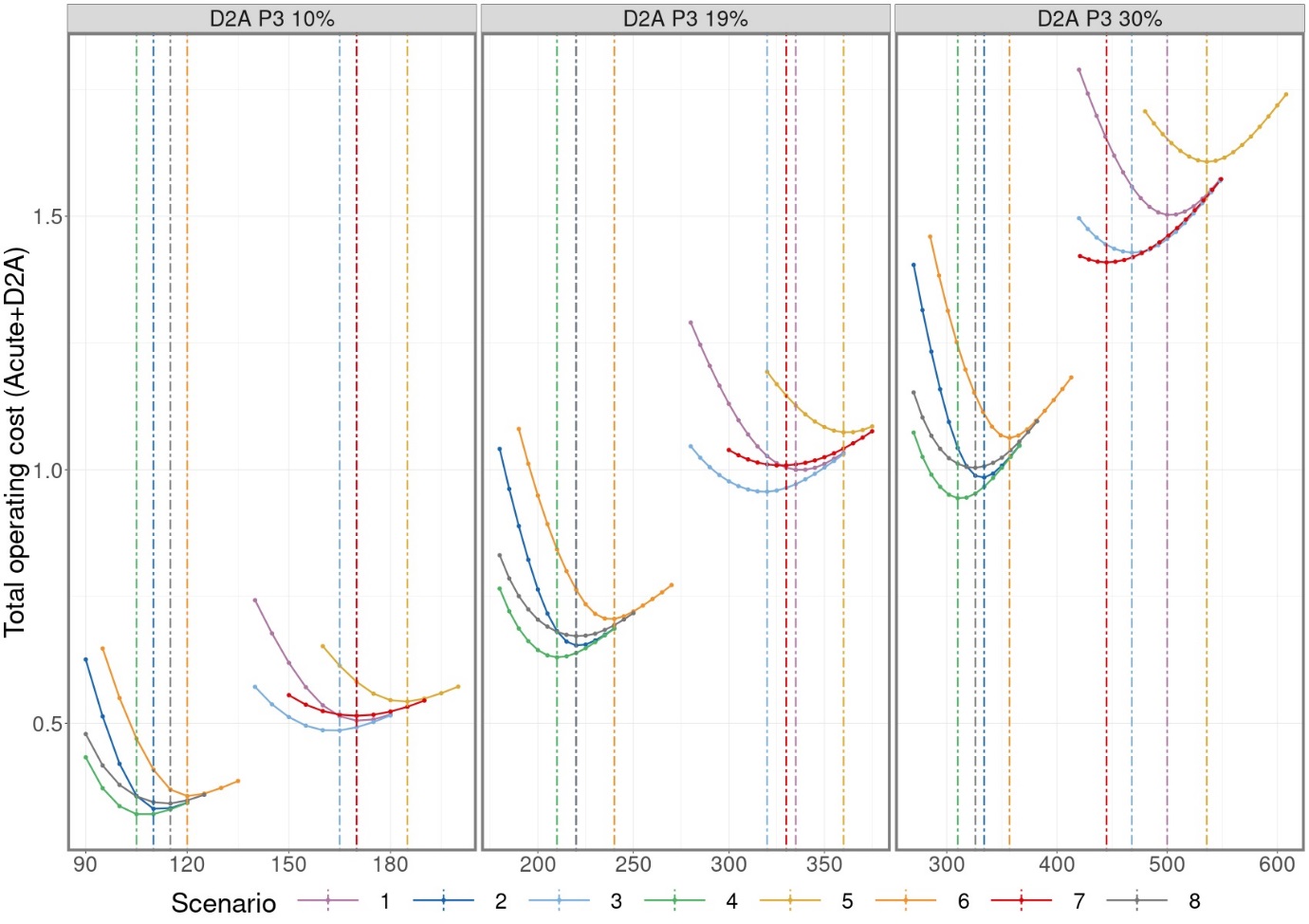


**Figure SI.2.3.** Weekly cost of acute delay and pathway care community service for capacity configurations for Pathway 3 with 10%, 19% (baseline) and 30% of acute admissions requiring complex community services at discharge.
