# Supplementary material for: Optimising the balance of acute and intermediate care capacity for the complex discharge pathway: Computer modelling study during COVID-19 recovery in England
Source: PLoS One. 2022 Jun 7;17(6):e0268837. doi: 10.1371/journal.pone.0268837 (PMC9173611; doi:10.1371/journal.pone.0268837)
Supplement: S3 File — (DOCX) [file pone.0268837.s003.docx]

**Supporting Information 3: Modelled results under assumption of no capacity constraint**


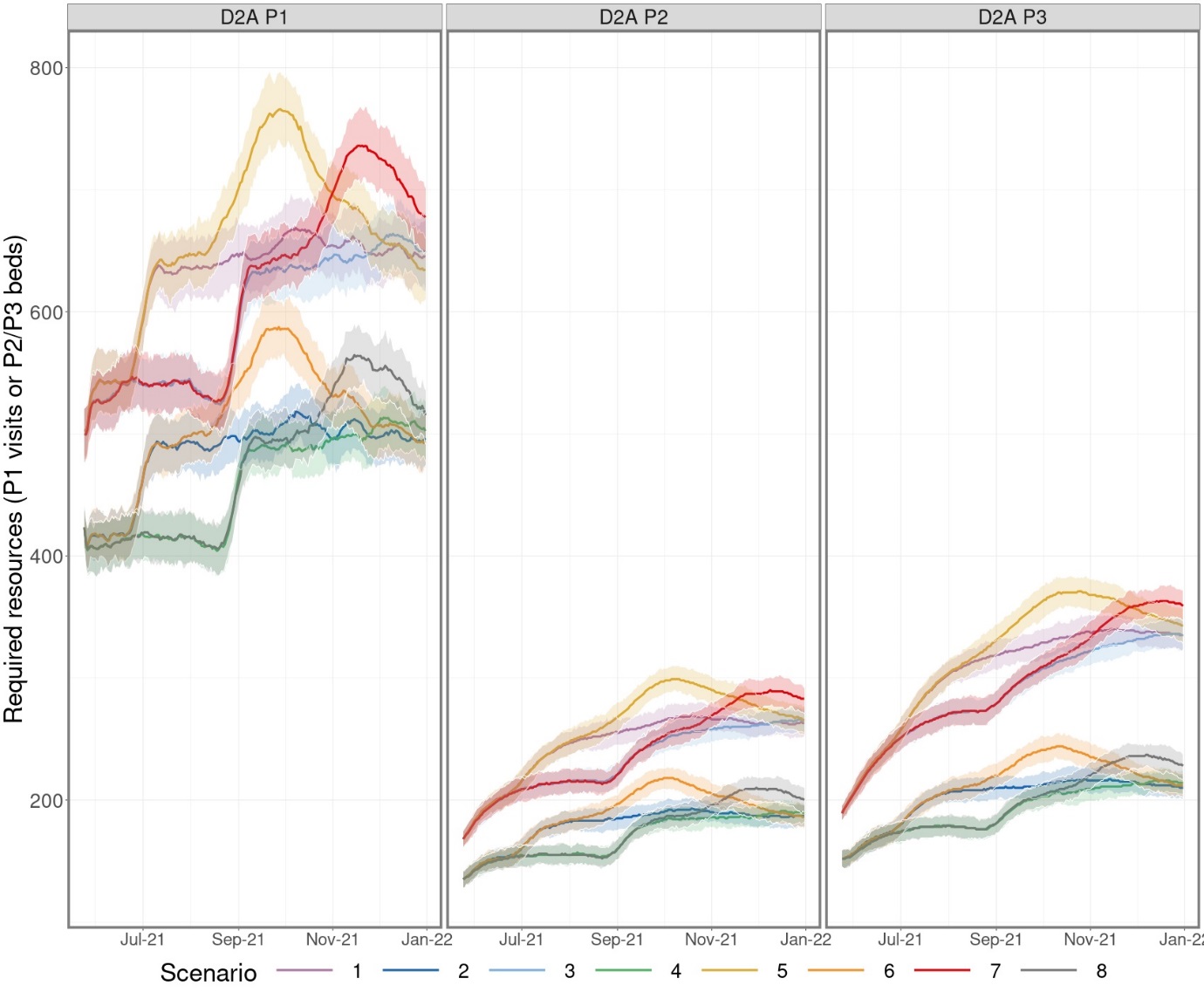


**Figure SI.3.1.** Projections of bed occupancy over time for considered scenarios under the assumption of no community services capacity constraint with interquartile range.
