# Supplementary material for: Optimising the balance of acute and intermediate care capacity for the complex discharge pathway: Computer modelling study during COVID-19 recovery in England
Source: PLoS One. 2022 Jun 7;17(6):e0268837. doi: 10.1371/journal.pone.0268837 (PMC9173611; doi:10.1371/journal.pone.0268837)
Supplement: S4 File — (DOCX) [file pone.0268837.s004.docx]

**Supporting Information 4: Research checklist (STRESS)**

**Strengthening the Reporting of Empirical Simulation Studies (STRESS)**

**Discrete-event simulation guidelines STRESS-DES**

**Paper title:** Optimising the balance of acute and intermediate care capacity for the complex discharge pathway: computer modelling study during COVID-19 recovery in England

| **Section/Subsection** | **Item** | **Recommendation** | | **Submitted paper** |
| --- | --- | --- | --- | --- |
| 1. **Objectives** |  |  | |  |
| Purpose of the model | 1.1 | Explain the background and objectives for the model. | | To estimate the theoretical cost-optimal capacity requirement for ‘step down’ intermediate care services within a major healthcare system in England, at a time when considerable uncertainty remained regarding vaccination uptake and the easing of societal restrictions |
| Model Outputs | 1.2 | Define all quantitative performance measures that are reported, using equations where necessary. Specify how and when they are calculated during the model run along with how any measures of error such as confidence intervals are calculated. | | Key simulation output measures of interest consist of: (i) Total cost of both intermediate care service provision (calculated from the modelled capacity levels) and the acute capacity required to support any delays to discharge (calculated from the mean number of acute beds blocked); (ii) Percentage occupancy per modelled capacity levels; (iii) Numbers of patients delayed per modelled capacity levels.  Each simulation was run until 31 December 2021, with 200 replications performed for each simulation in order to capture the realistic effect of variability (with respect to arrivals and lengths of stay). Results for each simulation were calculated from the outputs of these replications.  See also Methods section in paper and Supplementary Material A. |
| Experimentation Aims | 1.3 | If the model has been used for experimentation, state the objectives that it was used to investigate.     1. Scenario based analysis – Provide a name and description for each scenario, providing a rationale for the choice of scenarios and ensure that item 2.3 (below) is completed. 2. Design of experiments – Provide details of the overall design of the experiments with reference to performance measures and their parameters (provide further details in *data* below). 3. Simulation Optimisation – (if appropriate) Provide full details of what is to be optimised, the parameters that were included and the algorithm(s) that was be used. Where possible provide a citation of the algorithm(s). | | Scenario based analysis. Full details of scenarios included in Table 1 in the main paper with explanations as to why each is investigated provided in the referring section (subsection Scenario Analysis within Methods chapter). |
| 1. **Logic** |  |  | |  |
| Base model overview diagram | 2.1 | Describe the base model using appropriate diagrams and description. This could include one or more process flow, activity cycle or equivalent diagrams sufficient to describe the model to readers. Avoid complicated diagrams in the main text. The goal is to describe the breadth and depth of the model with respect to the system being studied. | | 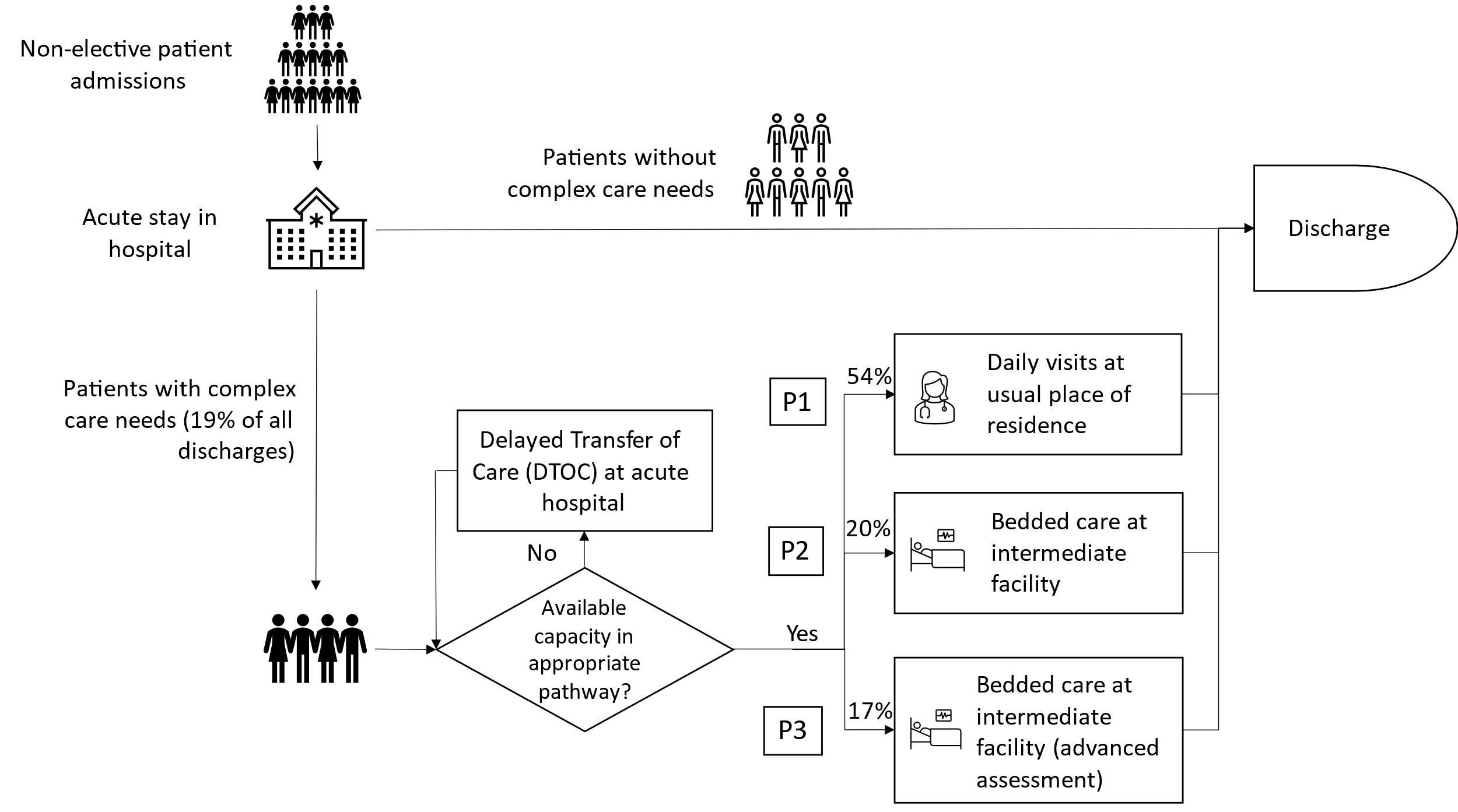 |
| Base model logic | 2.2 | Give details of the base model logic. Give additional model logic details sufficient to communicate to the reader how the model works. | | See section Computer simulation modelling in chapter Methods and Supplementary Material A. |
| Scenario logic | 2.3 | Give details of the logical difference between the base case model and scenarios (if any). This could be incorporated as text or where differences are substantial could be incorporated in the same manner as 2.2. | | The difference between the base case model and the scenarios is in the values of the input parameters (clearly described in Table 1 in the main paper). |
| Algorithms | 2.4 | Provide further detail on any algorithms in the model that (for example) mimic complex or manual processes in the real world (i.e. scheduling of arrivals/appointments/operations/maintenance, operation of a conveyor system, machine breakdowns, etc.). Sufficient detail should be included (or referred to in other published work) for the algorithms to be reproducible. Pseudo-code may be used to describe an algorithm. | | Implementation of this model is through the iterative three-phased method of discrete event simulation (Pidd, 1998).  Full details are provided in section Computer Simulation of the paper and Supplementary Material A. |
| Components | 2.5 | 2.5.1 Entities | Give details of all entities within the simulation including a description of their role in the model and a description of all their attributes. | Individual patients, each patient has an arrival time and a planned duration of care (either in bedded or visits based care) time as sampled from the appropriate length of stay distribution.  Full details are provided in section Computer Simulation of the paper and Supplementary Material A. |
|  |  | 2.5.2 Activities | Describe the activities that entities engage in within the model. Provide details of entity routing into and out of the activity. | Arrival (ready to be discharged from acute care hospital), start of service (visits or bedded depending on the pathway model) and end of service.  See Supplementary Material A for more details. |
|  |  | 2.5.3 Resources | List all the resources included within the model and which activities make use of them. | There are two types of resources depending on the pathway model: ‘slots’ for the visit-based care pathway model and ‘beds’ for the bedded care pathway model.  See Supplementary Material A for more details. |
|  |  | 2.5.4 Queues | Give details of the assumed queuing discipline used in the model (e.g. First in First Out, Last in First Out, prioritisation, etc.). Where one or more queues have a different discipline from the rest, provide a list of queues, indicating the queuing discipline used for each. If reneging, balking or jockeying occur, etc., provide details of the rules. Detail any delays or capacity constraints on the queues. | A simulated patient waiting to be scheduled in one of the three pathway models, is assumed to be occupying a bed in the acute care hospital.  Queue discipline is FIFO apart from the visit-based model (P1), where patients with a long duration of service, and/or a high initial/end visit requirement subsequently may prevent patients with lower service requirements from entering the P1 system. For this reason, if there are no available resources to start service for a new patient immediately, an arriving patient is scheduled to start on the following day. A patient whose visit sequence can be integrated into the available P1 capacity will be scheduled immediately.  See Supplementary Material A for more details. |
|  |  | 2.5.5 Entry/Exit Points | Give details of the model boundaries i.e. all arrival and exit points of entities. Detail the arrival mechanism (e.g. ‘thinning’ to mimic a non-homogenous Poisson process or balking) | Entry: patient ready to be discharged from acute care hospital requiring placement in one of the three pathways (P1, P2, P3)  Exit point: discharged from one of P1, P2 or P3 |
| 1. **Data** |  |  | |  |
| Data sources | 3.1 | List and detail all data sources. Sources may include:   - Interviews with stakeholders, - Samples of routinely collected data, - Prospectively collected samples for the purpose of the simulation study, - Public domain data published in either academic or organisational literature. Provide, where possible, the link and DOI to the data or reference to published literature.   All data source descriptions should include details of the sample size, sample date ranges and use within the study. | | All secondary datasets used in the simulation models were supplied by the Clinical Commissioning Group (BNSSG CCG) and intermediate care providers (Sirona). Lengths of Stay distributions for each pathway were fitted to data 17/04/2020 – 10/02/2021 inclusive. At the start of each simulation, the initial occupancy and waiting list size was set equal to their actual values as of 14 May 2021, as provided by the CCG. Proportions of complex discharges and proportions of patients entering each pathway were empirically derived in collaboration with the CCG and detailed in Table SI.1.1. Cost sources are detailed in Table SI.1.2. |
| Pre-processing | 3.2 | Provide details of any data manipulation that has taken place before its use in the simulation, e.g. interpolation to account for missing data or the removal of outliers. | | Demand projections are estimated from two different models, COVID-19 related demand from an SEIR model (Powell & Wood, 2021)., and the remaining demand from a regression model (Powell & Wood, 2021).  See sub-section Demand projection in the Methods section of the main paper. |
| Input parameters | 3.3 | List all input variables in the model. Provide a description of their use and include parameter values. For stochastic inputs provide details of any continuous, discrete or empirical distributions used along with all associated parameters. Give details of all time dependent parameters and correlation.  Clearly state:   - Base case data - Data use in experimentation, where different from the base case. - Where optimisation or design of experiments has been used, state the range of values that parameters can take.   Where theoretical distributions are used, state how these were selected and prioritised above other candidate distributions. | | See Tables SI.1.1 and 2 in the Supplementary Material A. |
| Assumptions | 3.4 | Where data or knowledge of the real system is unavailable what assumptions are included in the model? This might include parameter values, distributions or routing logic within the model. | | See assumptions in Methods section and limitations in the Discussion. |
| 1. **Experimentation** |  |  | |  |
| Initialisation | 4.1 | Report if the system modelled is terminating or non-terminating. State if a warm-up period has been used, its length and the analysis method used to select it. For terminating systems state the stopping condition.  State what if any initial model conditions have been included, e.g., pre-loaded queues and activities. Report whether initialisation of these variables is deterministic or stochastic. | | Non-terminating system. No warm-up period was used. Instead, at the start of each simulation, the initial occupancy and waiting list size was set equal to their actual values as of 14 May 2021.  Full details are provided in section Computer Simulation of the paper and Supplementary Material A. |
| Run length | 4.2 | Detail the run length of the simulation model and time units. | | Time unit is day. Simulation runs until 31 Dec 2021. |
| Estimation approach | 4.3 | State the method used to account for the stochasticity: For example, two common methods are multiple replications or batch means. Where multiple replications have been used, state the number of replications and for batch means, indicate the batch length and whether the batch means procedure is standard, spaced or overlapping. For both procedures provide a justification for the methods used and the number of replications/size of batches. | | 200 multiple replications were used for each scenario. |
| 1. **Implementation** |  |  | |  |
| Software or programming language | 5.1 | State the operating system and version and build number.  State the name, version and build number of commercial or open source DES software that the model is implemented in.  State the name and version of general-purpose programming languages used (e.g. Python 3.5).  Where frameworks and libraries have been used provide all details including version numbers. | | The model was coded from scratch in R and has been released as an open-source tool (hosted on <https://github.com/nhs-bnssg-analytics/ipacs-v1-model> and promoted via social media).  Model data available at <https://github.com/nhs-bnssg-analytics/c19-recovery-community-services-modelling> |
| Random sampling | 5.2 | State the algorithm used to generate random samples in the software/programming language used e.g. Mersenne Twister.  If common random numbers are used, state how seeds (or random number streams) are distributed among sampling processes. | | Uses the inbuilt random number generator in R. Each replication uses a different seed call to this function. This provides the necessary stochastic variation within each replication, yet also allows reproducible model scenarios to be created and assessed (useful when evaluating specific changes in the model parameters). |
| Model execution | 5.3 | State the event processing mechanism used e.g. three phase, event, activity, process interaction.  *Note that in some commercial software the event processing mechanism may not be published. In these cases authors should adhere to item 5.1 software recommendations.*  State all priority rules included if entities/activities compete for resources.  If the model is parallel, distributed and/or use grid or cloud computing, etc., state and preferably reference the technology used. For parallel and distributed simulations the time management algorithms used. If the HLA is used then state the version of the standard, which run-time infrastructure (and version), and any supporting documents (FOMs, etc.) | | In this study, ‘discrete time’ simulation was used to dynamically model the flow of individual patients from acute discharge readiness (i.e. to become an ‘arrival’ at the start of a D2A waiting list) through to completion of intermediate care. Essentially this involves simulating the arrival of simulated individual patients (see Method; Demand projection), commencement of intermediate care in the relevant D2A pathway (for which the patient may have to wait, depending on available capacity), and their departure from the service (determined by their length of stay).  A separate model was constructed for each of the three D2A pathways and the eight scenarios considered (Table 1). At the start of each simulation, the initial occupancy and waiting list size was set equal to their actual values as of 14 May 2021. Within each simulation, each future day (the ‘discrete time’ interval used in this study) was simulated consecutively, with instances of the above-mentioned three events performed in line with the simulation schedule (the arrivals onto the D2A pathway which were due to occur that day; how many patients were due to complete their D2A pathway that day; and the commencement of intermediate care provided there were patients waiting for care and available capacity).  More information in Supplementary Material A. |
| System Specification | 5.4 | State the model run time and specification of hardware used. This is particularly important for large scale models that require substantial computing power. For parallel, distributed and/or use grid or cloud computing, etc. state the details of all systems used in the implementation (processors, network, etc.) | | Processing time is insubstantial, typically taking less than five minutes for each scenario evaluated on a desktop computer. |
| 1. **Code Access** |  |  | |  |
| Computer Model Sharing Statement | 6.1 | Describe how someone could obtain the model described in the paper, the simulation software and any other associated software (or hardware) needed to reproduce the results. Provide, where possible, the link and DOIs to these. | | The tool is open source and available for free: <https://github.com/nhs-bnssg-analytics/ipacs-v1-model> |
